# Astragalar Morphology of Selected Giraffidae

**DOI:** 10.1371/journal.pone.0151310

**Published:** 2016-03-30

**Authors:** Nikos Solounias, Melinda Danowitz

**Affiliations:** 1 Department of Anatomy, New York Institute of Technology College of Osteopathic Medicine, Old Westbury, NY, United States of America; 2 Department of Paleontology, American Museum of Natural History, Central Park West at 79^th^ Street, New York, NY, United States of America; Institute of Vertebrate Paleontology and Paleoanthropology Chinese Academy of Sciences, CHINA

## Abstract

The artiodactyl astragalus has been modified to exhibit two trochleae, creating a double pullied structure allowing for significant dorso-plantar motion, and limited mediolateral motion. The astragalus structure is partly influenced by environmental substrates, and correspondingly, morphometric studies can yield paleohabitat information. The present study establishes terminology and describes detailed morphological features on giraffid astragali. Each giraffid astragalus exhibits a unique combination of anatomical characteristics. The giraffid astragalar morphologies reinforce previously established phylogenetic relationships. We find that the enlargement of the navicular head is a feature shared by all giraffids, and that the primitive giraffids possess exceptionally tall astragalar heads in relation to the total astragalar height. The sivatheres and the okapi share a reduced notch on the lateral edge of the astragalus. We find that *Samotherium* is more primitive in astragalar morphologies than *Palaeotragus*, which is reinforced by tooth characteristics and ossicone position. Diagnostic anatomical characters on the astragalus allow for giraffid species identifications and a better understanding of Giraffidae.

## Introduction

The artiodactyl astragalus is a remarkable adaptation, allowing for a specialized axis of motion. The astragalus is modified to possess two distinct trochleae; one proximally and the other distally at the head, creating a double-pullied structure. The proximal trochlea slides against the tibia, and the distal trochlea articulates with the cubonavicular. The artiodactyl astragalus is modified to allow for substantial dorso-plantar motion due partly to the expansion of the head into a trochlea, and limited mediolateral motion due to a central notch, or groove in the head [1]. This modified astragalus acts as a powerful cam-shaft, reinforcing dorsi-flexion and plantar-flexion and providing a powerful thrust to the foot.

Morphological features on mammalian astragali have been useful in the separation and identification of many higher-order groups. Astragalar features have been used to define Proboscidea, Archonta, and Artiodactyla [2–4]. Morphometric analyses of mammalian astragali, however, have been largely unsuccessful in separating species, but have greater utility in paleohabitat predictions [5,6]. Morphometric features of the astragalus can provide information on body size, as well as ecological habitat [7]. Astragalar studies therefore allow for the reconstruction of habitats and for the examination of ecological preferences [8]. Detailed descriptions of the morphology are not clearly translated into metric variables, but appear to have utility in systematics and species identifications. Thewissen and Madar (1999) demonstrated that astragalar features have a clear phylogenetic signal, especially when superimposed on previously established cladistic relationships [1].

The present study establishes astragalar terminology to allow for detailed anatomical descriptions and comparisons between species. Using this terminology, we describe the astragalar morphology of 12 species representing all 7 subfamilies of Giraffidae, as well as *Prodremotherium*, a potential ancestor of the family. The astragalus is a robust skeletal element that readily fossilizes and is abundant in many fossil collections [9]. Therefore, diagnostic characters of the astragalus would facilitate future species identifications and contribute to a better understanding of Giraffidae.

## Materials and Methods

**Fig 1:**
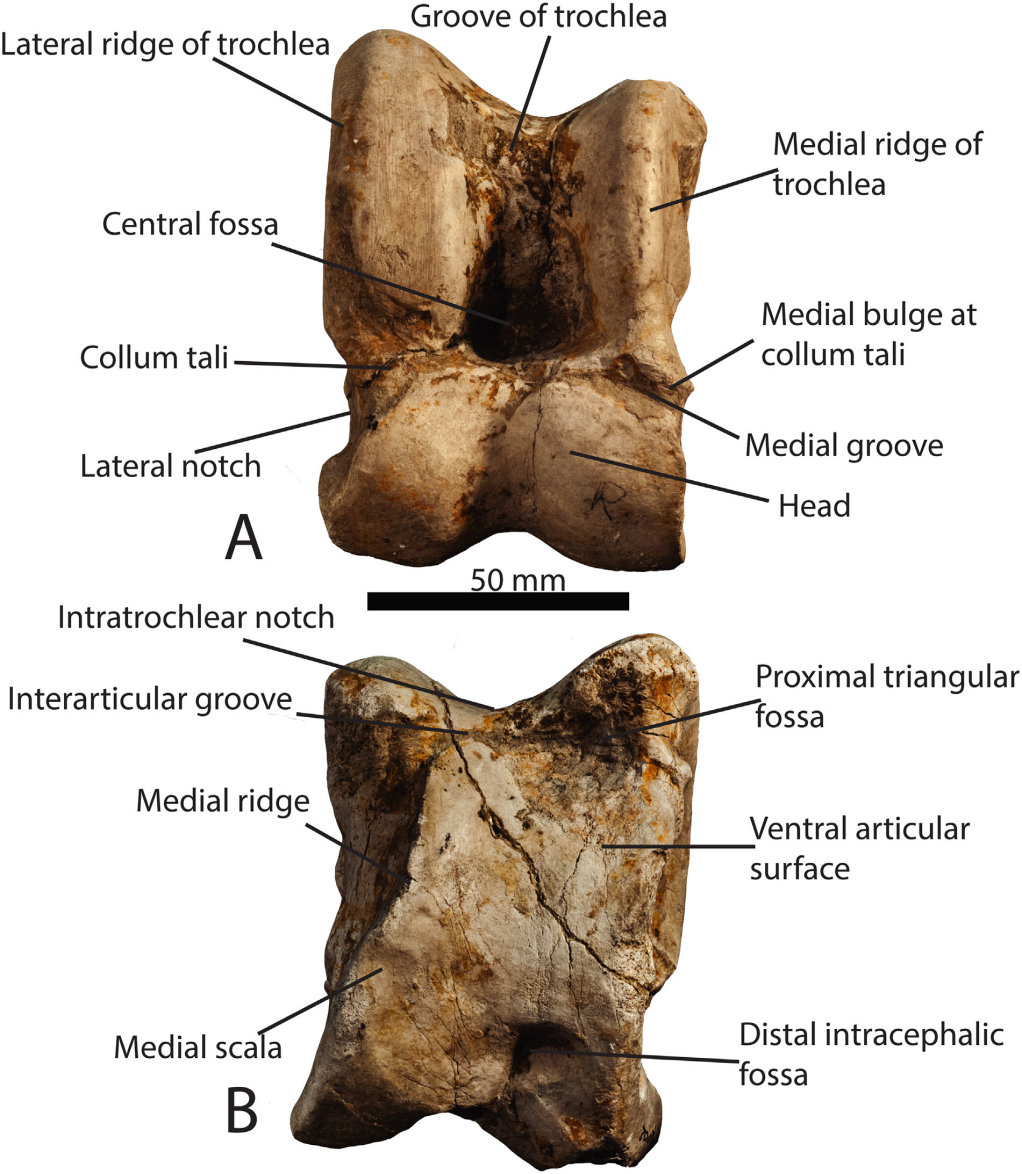
Astragalar terminology. (A) Photograph of a *Samotherium major* (GMM 2002) astragalus in dorsal view, with representative terminology. (B) *Samotherium major* (GMM 2002) astragalus in ventral view, with representative terminology. The scale bar represents 50 mm.

We identify the astragali of 12 giraffid species and *Prodremotherium* (Table 1). Identifications were based on known species identifications at each locality, as well as size differences between the taxa. We establish terminology (Fig 1), and describe the detailed morphological features of the astragalus of *Samotherium major*, because it is a representative giraffid species that exhibits a mosaic of primitive and specialized features. Subsequently, we compare each taxon against *S*. *major*. The astragalar specimens utilized for the descriptions were chosen from the type locality where each species was named. We measure the medial length, lateral length, and distal width using standard calipers in millimeters (Table 2). The astragali utilized for morphological descriptions and measurements are housed in the American Museum of Natural History, New York (AMNH), Geomuseum of the WWU, Münster (GMM), Geological Museum of Lausanne (MGL), Muséum National d’Histoire Naturelle, Paris (MNHN), Natural History Museum, Bern (NHM Be), Natural History Museum, London (NHM UK), Pakistan Natural History Museum, Islamabad (PMNH), Palaeontological Institute of Uppsala (PIU), University of Bristol (UB), University of California, Berkeley (UCB). All specimens were collected legally and have been housed in established natural history museums. All specimens are accessible to visiting scientists with permission from the curators.

**Table 1:** Giraffid astragali utilized in the morphological descriptions. Specimens in bold are represented in Figs 2 and 3.

Species	Specimen Numbers
*Samotherium major*	GMM 2001, **GMM 2002**, MGL 082805 (S594), MGL 082781 (S1015)
*Prodremotherium elongatum*	**AMNH 10339**
*Canthumeryx sirtensis*	**NHM UK (no number)**
*Giraffokeryx punjabiensis*	**AMNH 95155**, AMNH 19453, PNMH 24279
*Helladotherium duvernoyi*	**MNHN PIK1547**, NHM UK 11387, NHM UK 11388
*Bramatherium megacephalum*	**AMNH 19461**, PNMH 51840
*Sivatherium giganteum*	**NHM UK 86691**, NHM UK 988, PNMH 6216
*Okapia johnstoni*	AMNH 51903, AMNH 51220, AMNH 51196, AMNH 51218, AMNH 51198, **AMNH 51219**, AMNH 51221
*Palaeotragus rouenii*	**MNHN PIK1695**, MGL 492, MGL S673
*Bohlinia attica*	MNHN PIK1633, **MNHN PIK1634A**, MNHN PIK1634B, MNHN MAR647b
*Honanotherium schlosseri*	**PIU 3597**
*Giraffa camelopardalis*	**AMNH 82001**, AMNH 53550, AMNH 82003, AMNH 83458

**Table 2:** Selected measurements of giraffid astragali (in millimeters).

Species	Specimen #	Lateral length	Medial length	Distal width
*Samotherium major*	AMNH 19974	116.82	97.57	78.54
*Samotherium major*	AMNH 96122	108.36	92.52	69.79
*Samotherium major*	MGL 594	101.5	93	70
*Samotherium major*	MGL 1015	93	81	62
*Prodremotherium elongatum*	AMNH 10339a	27.14	26.32	15.03
*Prodremotherium elongatum*	AMNH 10339b	22.41	20.47	12.34
*Canthumeryx sirtensis*	UCB V4899/4258	39		25
*Canthumeryx sirtensis*	UCB V48100/41854	44		29
*Canthumeryx sirtensis*	UB 20120	49		29
*Canthumeryx sirtensis*	UB 20121	41		29
*Giraffokeryx punjabiensis*	AMNH 19453	57.72	54.61	31.40
*Giraffokeryx punjabiensis*	AMNH 1953	66.76	60.76	36.32
*Giraffokeryx punjabiensis*	AMNH 95155	59.43	54.09	35.52
*Giraffokeryx punjabiensis*	AMNH 19677	61.48	57.05	37.85
*Helladotherium duvernoyi*	NHM UK11388a	102	89.2	63.4
*Helladotherium duvernoyi*	NHM UK11388b	98	86.5	65
*Helladotherium duvernoyi*	NHM UK11389	98.9	93.8	71.1
*Helladotherium duvernoyi*	NHM UK11387	113.7	99.1	75.4
*Bramatherium megacephalum*	AMNH 19461	107.76	91.07	71.85
*Sivatherium giganteum*	NHM UK86691	105.91	124.68	106.12
*Sivatherium giganteum*	NHM UK998		130.27	113.23
*Okapia johnstoni*	AMNH 51120	67.18	58.38	45.31
*Okapia johnstoni*	AMNH 51218	69.32	59.87	47.58
*Okapia johnstoni*	AMNH 51198	72.22	62.34	48.03
*Okapia johnstoni*	AMNH 51196	70.3	58.49	45.15
*Paleotragus rouenii*	MGL 492	73		47.5
*Paleotragus rouenii*	MNHN PIK1695	71		45
*Bohlinia attica*	NHMBe **no number**	103	99	76.7
*Bohlinia attica*	NHMBe **no number**	105	94	70
*Bohlinia attica*	NHMBe **no number**	105	91	71
*Bohlinia attica*	NHMBe **no number**	108	94	73
*Honanotherium schlosseri*	AMNH **no number**	109	92	79
*Giraffa camelopardalis*	AMNH 53350	103.16	92.5	75.32
*Giraffa camelopardalis*	AMNH 82003	83.95	75.33	61.04
*Giraffa camelopardalis*	AMNH 83458	95.78	85.13	66
*Giraffa camelopardalis*	AMNH 80146	82.09	72.91	59.14

## Results

### General description of a giraffid astragalus

In dorsal view, the proximal portion of the astragalus is termed the trochlea, or the tibial trochlea. It is composed of a lateral ridge, and a slightly shorter medial ridge, separated by a depression termed the groove of the trochlea. The fibula and calcaneum articulate lateral to the lateral edge of the trochlea. The tibia articulates with the entire trochlea, and the tibial trochlea slides in the trochlear groove. At the distal aspect of the trochlea is the central fossa, which delineates the position of the tibial cochlea during maximal dorsi-flexion of the foot. Distal to the trochlea is the head of the astragalus; the head is separated from the trochlea by a neck, termed the collum tali. The head of the astragalus is separated into a medial and lateral bulge by a median depression or groove. The head articulates with the cubonavicular, and the median depression marks original fusion of the cuboid and navicular bones.

In ventral view, the majority of the surface articulates with the calcaneum. The proximal-most aspect of the medial and lateral edges of the trochlea are visible, and are separated by the intratrochlear notch. At the proximal edge, there is a triangular depression termed the proximal triangular fossa, located at the ventral aspect of the lateral edge of the trochlea. This marks maximal dorsiflexion between the astragalus and the calcaneum. Medial to the proximal triangular fossa, there is often a groove separating the ventral articular surface from the articular surface of the trochlea. We term this space the interarticular groove. On the medial edge, there is sometimes a step between the ventral articular surface and the medial aspect of the head, which we term the medial scala. There is an elevated ridge on the medial ventral surface, termed the medial ridge. The medial scala delineates maximal plantar-flexion between the astragalus and the cubonavicular. At the distal aspect of the ventral surface, there is a depression, often with two distinct areas, between the medial and lateral aspects of the head. We term this the distal intracephalic fossa, and like the medial scala, it marks maximal plantar-flexion between the astragalus and the cubonavicular. (Fig 1)

### Complete description of the *Samotherium major* astragalus

***Samotherium major***.

Specimens: GMM 2001, GMM 2002, MGL 082805 (S 594), MGL 082781 (S 1015)

Type locality: Samos

Age: 7.5 Ma

Subfamily: Palaeotraginae

**Fig 2:**
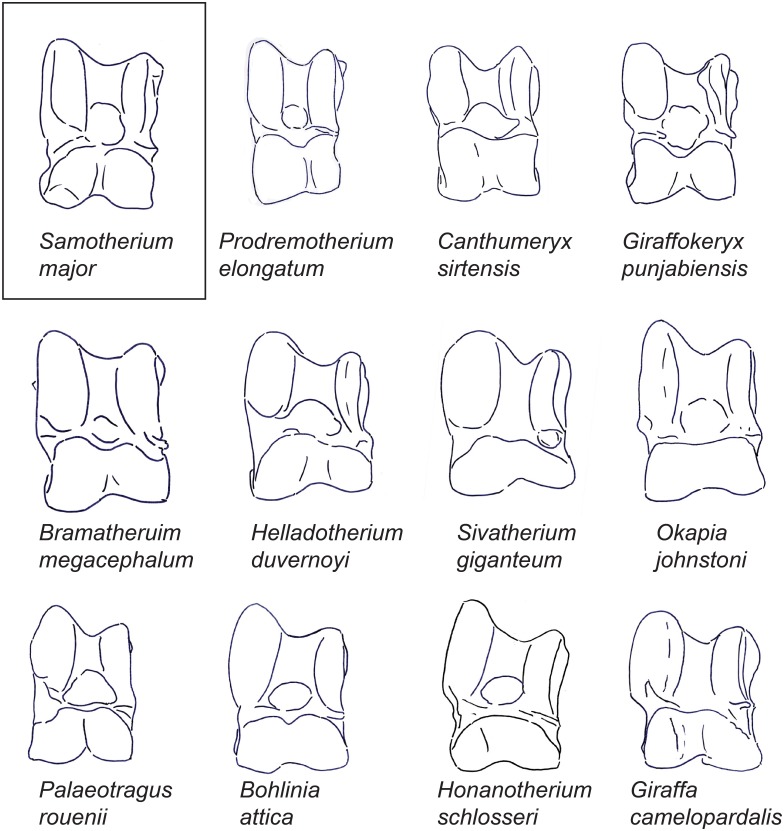
The astragali of representative giraffids in dorsal view. Demonstration of a dorsal view of representative astragali for all taxa evaluated in this study. Each specimen is isometrically scaled so that all specimens are of equal height. *Samotherium major* is represented in a box, as it is the baseline astragalus from which all taxa were subsequently compared against.

In dorsal view, the lateral proximal edge of the trochlea is notably taller and thicker than the medial edge. The lateral edge of the trochlea is straight. There is a faint groove on the dorsal surface of the lateral ridge of the trochlea. The central fossa is large and shallow. The trochlea is slightly twisted laterally in relation to the head of the astragalus. The groove of the trochlea is flattened. There is a protruding, pointed bulge on the medial surface of the collum tali, and the lateral collum tali is flat. The proximal edge of the articular surface of the head is flat with a slight central depression, and it has a vertical edge medially and a deep slant laterally. The medial aspect of the head is more massive than the lateral side. There is a deep, wide groove running obliquely between the medial head and the medial collum tali, following the synovial cavity surface. The lateral edge of the astragalus is notched between the trochlea and the head. The head is notched distally, creating a distinct medial and lateral bulge. The astragalus is narrow and rectangular shaped. (Fig 2)

**Fig 3:**
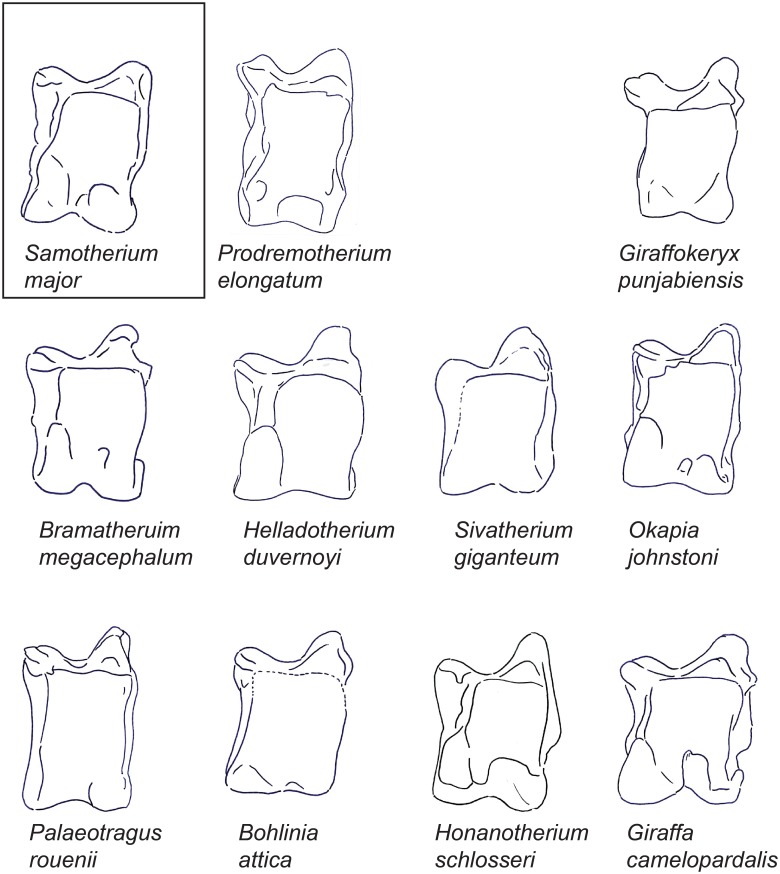
The astragali of representative giraffids in ventral view. Demonstration of a ventral view of representative astragali for all taxa evaluated in this study. Each specimen is isometrically scaled so that all specimens are of equal height. *Samotherium major* is represented in a box, as it is the baseline astragalus from which all taxa were subsequently compared against.

In ventral view, there is a distinct notch on the lateral aspect of the proximal-lateral trochlea. The intratrochlear notch is wide, and the proximal triangular fossa is shallow. The interarticular groove is narrow, and it is continuous laterally with the proximal triangular fossa. The ventral articular surface of the medial trochlea is lipped and is oriented obliquely. The ventral articular surface has a medial ridge that is directed towards the intratrochlear notch. There is a faint medial scala. The distal intracephalic fossa has two distinct areas; there is a prominent lateral area and a very faint medial area slightly displaced distally. (Fig 3)

In medial view, there is a deep groove proximally for the tibia. There is a pit at the distal surface that is intermediate in size and depth. The medial surface is separated from the ventral articular surface by a deep trough. In lateral view, there is a proximal protrusion that articulates with the fibula, which is pointed. The proximo-ventral facet for the calcaneum is small. There is a facet distally which articulates with the calcaneum, which is circular shaped.

### Selected giraffid astragali compared against *Samotherium major*

***Prodremotherium elongatum***.

Specimen: AMNH 10339

Type locality: Quercy

Age: 25 Ma

In dorsal view, the lateral ridge of the trochlea is only slightly taller than the medial ridge. The trochlea is notably twisted in relation to the head. The proximal edge of the articular surface of the head is straight with a central depression, and with vertical lateral and medial edges. The medial aspect of the head is fuller, and the lateral aspect of the head is notably wider. The obliquely oriented groove between the medial collum tali and the medial head is very faint and narrow. There is a very small notch on the lateral edge of the astragalus between the trochlea and the head. The astragalus is notably tall. (Fig 2)

In ventral view, there is no discernible notch on the lateral aspect of the proximal trochlea. The intratrochlear notch is relatively narrow, and there is no distinct proximal triangular fossa. The medial ridge of the ventral surface is directed towards the medial trochlea, creating a full and square shaped articular surface. There is a distinct medial scala, which is displaced distally. The distal intracephalic fossa presents as one unified, notably wide, shallow area at the distal ventral articular surface. (Fig 3)

In medial view, the proximal groove for the tibia is notably wide. The medial surface is separated from the ventral articular surface by a shallow trough. In lateral view, the proximal protrusion that articulates with the fibula is small.

***Canthumeryx sirtensis***.

Specimen: NHM UK no number

Type locality: Gebel Zelten

Age: 16 Ma

Subfamily: Canthumerycinae

In dorsal view, the lateral proximal edge of the trochlea is subequal in height with the medial edge. The lateral edge of the trochlea is arched. The trochlea is notably twisted in relation to the head of the astragalus. The proximal edge of the articular surface of the head is relatively straight, with vertical lateral and medial edges. The lateral aspect of the head is more massive than the medial aspect. The obliquely oriented groove between the medial collum tali and the medial head is notably faint and narrow. The lateral edge of the astragalus is slightly notched between the trochlea and the head. The head is notably tall in relation to the total height of the astragalus. (Fig 2)

***Giraffokeryx punjabiensis***.

Specimens: AMNH 95155, AMNH 19453, PNMH 24279

Type locality: Chinji

Age: 14 Ma

Subfamily: Giraffokerycinae

In dorsal view, the groove on the dorsal surface of the lateral ridge of the trochlea is absent. The proximal edge of the articular surface of the head has a large central depression, and vertical lateral and medial edges. The medial and lateral aspects of the head of the astragalus are subequal, with the lateral aspect being slightly larger. The groove between the medial head and medial collum tali is horizontally oriented. Instead of the typical notch, there is a wide groove at the center of the head, creating distinct lateral and medial bulges. The head of the astragalus is notably large when compared to the total height of the astragalus. (Fig 2)

In ventral view, the proximal triangular fossa is deep. The interarticular groove is notably wide. The ventral surface has a medial ridge that is directed vertically towards the medial proximal trochlea, creating a square shaped articular surface. There is a discernible medial scala. (Fig 3)

In medial view, the proximal groove for the tibia is wide. The distal pit is notably deep. There is no discernible trough separating the medial surface from the ventral articular surface.

***Bramatherium megacephalum***.

Specimens: AMNH 19461, PNMH 51840

Type locality: Upper Siwaliks

Age: 5 Ma

Subfamily: Sivatheriinae

In dorsal view, there is no discernible groove on the lateral ridge of the trochlea. The trochlea and the head of the astragalus are in the same plane. The proximal edge of the articular surface of the head is flat with a central depression, and a shallow slant medially and a vertical edge laterally. The lateral aspect of the head is taller, whereas the medial aspect of the head is wider. The lateral edge of the astragalus is slightly notched between the trochlea and the head. (Fig 2)

In ventral view, the notch on the lateral aspect of the proximal trochlea is distinct, and the proximal triangular fossa is deep. The medial ridge of the ventral surface is oriented vertically towards the medial trochlea, creating a square shaped articular surface, however, there is an indentation around the area of the medial scala, creating a discontinuity in the medial edge. The medial scala is present, and the distal intracephalic fossa is comprised of one distinct area. (Fig 3)

In lateral view, the proximal protrusion that articulates with the fibula is large and rounded. The proximo-ventral facet for the calcaneum is intermediate in size. The distal facet for the calcaneum is oval-shaped.

**Helladotherium duvernoyi**

Specimens: MNHN PIK1547, NHM UK 11387, NHM UK 11388

Type locality: Pikermi

Age: 7.5 Ma

Subfamily: Sivatheriinae

In dorsal view, the lateral ridge of the trochlea is exceptionally thick, and the medial ridge of the trochlea is notably thin. The lateral edge of the trochlea is slightly curved. The bulge at the medial collum tali is rounded. The proximal edge of the articular surface of the head is slanted medially, and has a sharper slant at the lateral edge. The lateral aspect of the head is more massive than the medial aspect. There is no notch at the lateral astragalus between the head and the trochlea. The astragalus is boxy shaped. (Fig 2)

In ventral view, the notch on the lateral aspect of the proximal trochlea is reduced. The medial ridge of the ventral articular surface is oriented between the medial trochlea and the intratrochlear notch, however there is an indentation proximal to the medial scala, creating a distinct discontinuity in the medial edge. The distal intracephalic fossa is notably faint to absent, and when present, it exhibits one discernible area. (Fig 3)

In medial view, the proximal groove for the tibia is notably deep. The distal pit is broad, and the medial surface is separated from the ventral articular surface by a shallow trough. In lateral view, the proximal protrusion for the fibula is large and rounded. There is no proxmo-ventral facet for the calcaneum. The distal facet for the calcaneum is circular shaped and protruding.

***Sivatherium giganteum***.

Specimens: NHM UK 86691, PNMH 6216, NHM UK 998

Type locality: Upper Siwaliks

Age: 2–3 Ma

Subfamily: Sivatheriinae

In dorsal view, the lateral ridge of the trochlea is exceptionally thick, and the medial ridge of the trochlea is notably thin. The lateral edge of the trochlea is slightly arched. The trochlea is notably twisted in relation to the head of the astragalus, and the central fossa is irregularly shaped. The proximal edge of the articular surface of the head forms a wide arc, with slight slanting at the medial and lateral edges that join centrally. The groove between the medial head and medial collum tali is narrow. There is a very slight notch on the lateral edge of the astragalus, between the head and the trochlea. The astragalus is boxy shaped, and is notably wide. (Fig 2)

In ventral view, the notch on the lateral aspect of the proximal trochlea is absent, and the ventral articular surface of the medial trochlea is not lipped. The medial ridge on the ventral articular surface is directed between the intratrochlear notch and the medial trochlea. There is no discernible medial scala nor distal intracephalic fossa. (Fig 3)

In medial view, the distal pit is notably large. The trough separating the medial surface from the ventral articular surface is very large and wide. In lateral view, the proximal protrusion that articulates with the fibula is large.

***Okapia johnstoni***.

Specimens: AMNH 51903, AMNH 51220, AMNH 51196, AMNH 51218, AMNH 51198, AMNH 51219, AMNH 51221

Subfamily: Okapiinae

In dorsal view, in place of the groove on the lateral ridge of the trochlea, there is a slight bony elevation. The trochlea and the head of the astragalus are in the same plane. The groove of the trochlea is notably flat, and the central fossa is irregularly shaped. The proximal edge of the articular surface of the head is flat with a deep slant laterally and a vertical edge medially. The groove between the medial head and the medial collum tali is horizontally oriented and shallow. There is a very slight notch between the trochlea and the head. (Fig 2)

In ventral view, the notch on the lateral aspect of the proximal trochlea is absent. The interarticular groove is notably wide and it is distinct laterally from the proximal triangular fossa. The medial ridge of the ventral articular surface is oriented vertically towards the medial trochlea, creating a square shaped ventral articular surface, however, there is an indentation around the area of the medial scala, creating a discontinuity in the edge. There is a distinct medial scala. The distal intracephalic fossa presents as one large, continuous area with a distinct depression in the center. (Fig 3)

In medial view, the proximal groove for the tibia is shallow. The medial surface is separated from the ventral articular surface by a shallow trough. In lateral view, the proximal protrusion that articulates with the fibula is small. The proximo-ventral facet for the calcaneum is elongated and large. The distal facet for the calcaneum is well-developed and oval shaped.

***Palaeotragus rouenii***.

Specimens: MNHN PIK1695, MGL 492, MGL S673

Type locality: Pikermi

Age: 7.5 Ma

Subfamily: Palaeotraginae

In dorsal view, the lateral edge of the trochlea is arched, and the central fossa is large and deep. The proximal edge of the articular surface of the head has a slant on the medial edge, a shallow depression centrally, and an abrupt step-down, forming an angle laterally. The groove between the medial head and medial collum tali begins narrow, and becomes wider by the medial edge of the astragalus. The lateral edge of the astragalus is slightly notched between the trochlea and the head. (Fig 2)

In ventral view, the notch on the lateral aspect of the proximal trochlea is reduced, and the intratrochlear notch is narrow. The ventral surface has a medial ridge that is directed medially towards the medial trochlea, creating a square-shaped ventral articular surface. The medial scala is faint, and there is no discernible distal intracephalic fossa. (Fig 3)

In medial view, the proximal protrusion for the fibula is small and rounded. The proximo-ventral facet that articulates with the calcaneum is elongated and large. The distal facet is oval shaped and large. In lateral view, the proximal groove for the tibia is notably deep. There is no trough separating the medial surface from the ventral articular surface.

***Bohlinia attica***.

Specimens: MNHN PIK1633, MNHN PIK1634A, MNHN PIK1634B, MNHN MAR647b

Type locality: Pikermi

Age: 7.5 Ma

Subfamily: Bohlininae

In dorsal view, the groove on the dorsal surface of the lateral ridge of the trochlea is absent. The central fossa is wide, and the lateral edge of the trochlea is arched. The trochlea and the head of the astragalus are in the same plane. The bulge on the medial surface of the collum tali is rounded and dorsally positioned, and the lateral collum tali is slightly bulging. The obliquely oriented groove between the medial collum tali and the medial head is very faint and narrow. The proximal edge of the articular surface of the head has a deep slant medially and a vertical edge laterally. The astragalus boxy shaped. (Fig 2)

In ventral view, the notch on the lateral aspect of the proximal trochlea is reduced. The proximal triangular fossa is shallow. The intratrochlear notch is somewhat narrow, and there is no discernible interarticular groove. The ventral surface has a medial ridge that is directed medially towards the medial proximal trochlea, creating a square shaped articular surface. The ventral surface is notably full. The medial scala is faint and the distal intracephalic fossa is small and displaced distally. (Fig 3)

In medial view, the distal pit is broad and expanded. In lateral view, there are two, dull proximal protrusions for the fibula, which are separated by a small groove. The proximo-ventral facet for the calcaneum is intermediate sized. The distal facet for the calcaneum is elongated and oval shaped.

***Honanotherium schlosseri***.

Specimens: PIU 3597

Type locality: Honan

Age: 7.5 Ma

Subfamily: Bohlininae

In dorsal view, the lateral edge of the trochlea is arched. The groove of the trochlea is notably flat. The groove between the medial head and medial collum tali begins narrow, and becomes wider by the medial edge of the astragalus. The proximal edge of the articular surface of the head forms an arc centrally, with a deep slant medially and a more vertical edge laterally. The collum tali is notably tall. The astragalus is boxy shaped. (Fig 2)

In ventral view, the notch on the lateral aspect of the proximal trochlea is absent. The interarticular groove is narrow, and it is distinct laterally from the proximal triangular fossa. The medial ridge of the ventral articular surface is oriented between the medial trochlea and the intratrochlear notch, however there is an indentation proximal to the medial scala, creating a distinct discontinuity in the medial edge. There is a distinct medial scala. The distal intracephalic fossa is large and square shaped. (Fig 3)

In medial view, the proximal groove for the tibia is notably deep. The distal pit is confined and deep. In lateral view, the proximal protrusion for the fibula is small and rounded. The proximo-ventral facet for the calcaneum is elongated and large. The distal facet is oval shaped, and the lateral surface of the trochlea is rounded.

***Giraffa camelopardalis***.

Specimens: AMNH 82001, AMNH 53550, AMNH 82003, AMNH 83458

Subfamily: Giraffinae

In dorsal view, the lateral edge of the trochlea is curved. In place of the groove on the dorsal surface of the lateral ridge of the trochlea, there is a slight bony elevation. The groove of the trochlea is exceptionally flat, and it is not distinct from the central fossa. The trochlea is notably twisted in relation to the head of the astragalus. The proximal edge of the articular surface of the head has a slight depression centrally, and is vertical laterally and deeply slanted medially. The medial aspect of the head is significantly larger than the lateral aspect. The collum tali is notably tall. The groove between the medial head and medial collum tali begins narrow, and becomes wider by the medial edge of the astragalus. There is a significant notch on the lateral edge of the astragalus, between the trochlea and the head. The distal notch on the head is large, creating a distinct lateral and medial bulge. Due to this notch, the ventral aspect of the head is visible dorsally. The astragalus is boxy shaped. (Fig 2)

In ventral view, the interarticular groove is not continuous with the proximal triangular fossa. The medial ridge of the ventral surface is oriented vertically towards the medial trochlea, creating a square shaped ventral articular surface. There is a faint medial scala, and a faint additional depression adjacent to it laterally. The distal intracephalic fossa presents as one notably deep area. (Fig 3)

In medial view, the distal pit is large. The proximal groove for the tibia is shallow. In lateral view, the proximal protrusion that articulates with the fibula is notably large and pointed. The distal facet for the calcaneum is triangular shaped. The lateral surface of the trochlea is rounded.

## Discussion

The giraffid astragali yield important diagnostic information, and the examination of morphological features can facilitate species descriptions. While each anatomical feature is often shared between several taxa, each individual species exhibits a unique combination of characteristics that allows for the identification of a species based on the astragalus. The complete astragalus is often well preserved in fossil collections [9], therefore the understanding of the morphological features that define each giraffid astragalus allows for the creation of faunal lists and the study of phylogenetic relationships.

The astragalar anatomy of giraffids closely resembles that of other large bodied ruminants, including bovids, cervids, and antilocaprids. These groups possess a central groove in the tibial trochlea as well as in the head, creating the characteristic artiodactyl double-pullied structure [10]. The trochlea and the head are relatively equal in width, and are separated by a short neck. The tibial trochlea is slightly taller than the head. In ventral view, the sustentacular facet comprises the majority of the ventral surface, unlike certain primitive artiodactyls such as *Diacodexis* [1], and there is a deep intratrochlear notch. The only feature separating giraffid astragali from other ruminants is the larger size. While our study is limited to giraffids, the morphological features described can be used to examine and compare other ruminant taxa.

*Prodremotherium elongatum* has been suggested as a plausible ancestor of Giraffidae. This taxon shares several characteristics with giraffids, such as the presence of elongated and proximally fused metapodials, small upper canines, and a reduced cingulum on the upper molars [11]. This taxon also shares the presence of strong metastylids with giraffids [12]. We note a distinct astragalar feature that is shared by all giraffids, but is absent in *Prodremotherium*. In the giraffids, we observe an enlargement of the navicular (medial) aspect of the head, whereas in *Prodremotherium*, the medial portion of the head of the astragalus is smaller. The *Prodremotherium* astragalus is notably elongated proximo-distally, a feature also seen in Tragulidae. In addition to the differences in the astragalus, Giraffidae are united by the biloboed canine, presence of ossicones, open ethmoidal fissure, absence of upper canines, and notable size increase [13]. The astragalar features that differ from *Prodremotherium* are among few non-cranial characteristics that can be used to define Giraffidae.

*Canthumeryx sirtensis* is the earliest giraffid known; it exhibits several primitive features such as an open nasolacrimal canal, a protruding occipital [14], and non-fused frontal bones at midline. *Giraffokeryx punjabiensis* is also primitive with its posteriorly positioned median palatine indentation, and non-fused frontals, but is more advanced in the position of its ossicones and the closure of the nasolacrimal canal [13,15]. *Prodremotherium* and *Canthumeryx*, share features of general neck elongation, where both taxa exhibit partially elongated cervical vertebrae [16]. All three of these taxa also share the flattened cranial bulge in the cervical vertebrae. We find several astragalar features that are shared in these primitive giraffids, as well as *Prodremotherium*. These taxa exhibit twisting of the trochlea in relation to the head, so that the medial side of the astragalus is partially visible adjacent to the trochlea in dorsal view. The astragalar head of these taxa is notably tall in relation to the total height of the astragalus. The proximal edge of the articular surface of the head is relatively straight centrally, with vertical lateral and medial edges. In *Canthumeryx* and *Prodremotherium*, there is a lessened height difference between the medial and lateral trochleae. *Giraffokeryx* is more advanced in this feature, and exhibits a significant height difference, typical of the other giraffids. Our astragalar features reinforce the previously suggested phylogenetic relationships, where *Prodremotherium* and *Canthumeryx* are primitive taxa, and *Giraffokeryx* exhibits some primitive features but is more advanced overall.

The sivatheres are a group of exceptionally large giraffids, united by the presence of two pairs of ossicones and a short diastema between the canine and the premolar [13,14,17]. The sivatheres included in this study are *Helladotherium*, *Bramatherium*, and *Sivatherium*. The okapi is similar to the sivatheres in having a short neck length, likely due to secondary shortening of the cervical vertebrae [16]. *Bramatherium* is more primitive as it retains the lateral notch between the head and the trochlea, which is common in Giraffidae; in the okapi, *Helladotherium*, and *Sivatherium*, the notch is notably reduced or absent. *Helladotherium* and *Sivatherium* share the feature where the lateral ridge of the trochlea is notably thick, and the medial ridge of the trochlea is notably thin. The central fossa is irregularly shaped in the okapi and *Sivatherium*. In the okapi, *Bramatherium*, and *Helladotherium*, there is an indentation on the medial edge of the ventral articular surface, proximal to the medial scala. The notch on the lateral aspect of the proximal trochlea, seen in ventral view, is largely reduced in *Helladotherium*, and is absent in the okapi and *Sivatherium*. At the present time, we do not resolve the specific interrelations between the individual sivatheres and the okapi based on the astragalar or previously established morphologies.

Members of Bohlininae are commonly referred to as the closet extinct relatives to Giraffinae. The taxa of Bohlininae included in this study are *Bohlinia* and *Honanotherium*. These two taxa share with the giraffe the presence of club-like ossicones, elongated metapodials, and p4 anterior cuspids directed mesiodistally [13,14]. In these taxa, the lateral edge of the trochlea is arched, creating a “cocked” appearance of the astragalus. These taxa also share the general morphology of the proximal articular surface of the head, where it appears flat laterally and slanted medially. In addition, the medial edge of the ventral articular surface of *Bohlinia* and the giraffe astragalus is directed medially, creating a square shaped articular surface, and both taxa possess a faint medial scala. The groove of the trochlea is exceptionally wide and flat, and the collum tali is notably tall in the astragali of *Honanotherium* and the giraffe. We find several astragalar similarities between the giraffe and members of Bohlininae, which reinforce their close phylogenetic affinity.

*Samotherium* and *Palaeotragus* are common late Miocene taxa with intermediate neck lengths, bare ossicones with wear facets, and notably small frontal sinuses [13,16,18,19]. Mitchell and Skinner (2003) suggested that *Samotherium* is more specialized than *Palaeotragus* based on hypsodonty and cranial sinuses [20]. Other studies, however, proposed that *Palaeotragus* is more specialized, with the p4 distal cuspids being as large as the medial cuspids, and having slender and elongated metapodials [13,14]. *Samotherium* is also more primitive in the position of the ossicones on the postero-lateral orbital margin [13]. We presently describe astragalar morphologies that reinforce this notion. In *Samotherium*, there is a smaller height difference between the medial and lateral trochlea, which more closely resembles *Prodremotherium* and *Canthumeryx*. *Palaeotragus* is more specialized in having a reduced lateral notch between the head and the trochlea, which is similar to the sivathere condition. *Palaeotragus* also has a ventral articular surface that is larger and square shaped, resembling that of *Bohlinia* and *Giraffa*. Based on the astragalar features, we agree with Hamilton (1978) and Solounias (2007) that *Palaeotragus* is a more specialized taxon than *Samotherium* [13,14].

We find that *Giraffa*, *Helladotherium*, *Sivatherium*, *Honanotherium*, and *Bohlinia* have the most relatively boxy astragali, when compared to the typical narrow-rectangular shape seen in the majority of the other giraffids. These taxa also represent the largest giraffids. We suggest that the unique astragalar dimensions in these taxa are shaped to accommodate the larger body size, and that a wider astragalus can better sustain the body weight on the limb. Conversely, *Prodremotherium* is the smallest of all the taxa evaluated, and this species has a notably long astragalus. It has been previously demonstrated that that bovid astragali dimensions positively correlated with body size [6]. It is also possible that there was a convergence in the astragalar shape of *Bohlinia* and *Honanotherium* with that of *Sivatherium and Helladotherium*, likely relating to locomotory differences.

Previous studies have demonstrated the relationship between astragalar morphometrics and the habitat preference of the species [5,7,8]. In our study, *Helladotherium duvernoyi*, *Samotherium major*, *Palaeotragus rouenii*, *Bohlinia attica*, and *Honanotherium schlosseri* all lived in the sclerophyllous evergreen woodland of Pikermi. We find, however, few morphological features shared by all Pikermian giraffids. In all taxa except *Samotherium major*, the lateral edge of the trochlea is arched, creating a “cocked” appearance of the astragalus; this feature is prominent in *Bohlinia attica* and *Honanotherium schlosseri*. In addition, the majority of these taxa possess a small or faint distal intracephalic fossa. We believe a more comprehensive study of giraffid limbs is needed to evaluate anatomical patterns in relation to paleohabitat.

It has been well studied that ecological substrates influence the functional morphology of post-cranial characters, notably in the limbs, which interact most directly with the environment [5,21]. Correspondingly, limb skeletal specimens, including the astragalus, provide information about the habitat preferences of an individual. The evolutionary history of a species, however, also plays a key role in shaping the anatomical features of the limbs [5]. The functional morphology therefore likely represents a mosaic of features shaped both by the phylogenetic history and environment of a species. DeGusta and Vrba (2003) and Davis and Calède (2012) apply strictly metric values to predict environmental preferences and to test species differences [5,6]. Our morphological features analyze astragalar characteristics of discrete aspects of the bone, which are not detected in broader metric comparisons. We believe that astragalar morphologies have utility in separating and identifying species, especially when relating these features to previously established relationships.
